# Gut microbiome dysregulation is associated with segmental glomerulosclerosis in IgA nephropathy: insights from Oxford classification-based microbiome profiling

**DOI:** 10.3389/fcimb.2026.1644626

**Published:** 2026-02-20

**Authors:** Bin Lu, Aiping Zhang, Mengqi Wu, Saiping Chen, Yuqing Wang, Jiarui Wang, Min Huang, Yanqin Zhu, Hong Liu, Fenggui Zhu, Xueyan Zeng, Shilei Chen, Xin Zhou, Riyang Lin

**Affiliations:** 1 Department of Nephrology, Hangzhou Traditional Chinese Medicine Hospital of Zhejiang Chinese Medical University, Hangzhou, Zhejiang, China; 2 Department of General Medicine, Xingqiao Street Community Health Service Center, Hangzhou, Zhejiang, China; 3 Department of Nephrology, Sichuan Second Hospital of Traditional Chinese Medicine, Chengdu, Sichuan, China; 4 Department of General Medicine, Community Health Service Centre, Hangzhou, Zhejiang, China; 5 Department of General Medicine, Tianshui Wulin Street Community Heal Care Centre, Hangzhou, Zhejiang, China; 6 Key Laboratory of Kidney Disease Prevention and Control Technology, Hangzhou, Sichuan, China

**Keywords:** 16S rRNA sequencing, gut microbiota, IgA nephropathy, Oxford classification, segmental glomerulosclerosis

## Abstract

**Background:**

IgA nephropathy (IgAN) is a common immune-complex-mediated glomerulonephritis with segmental glomerulosclerosis (S lesion, S1 in Oxford classification) being an independent predictor of poor renal prognosis, where 20%–40% of IgAN-S1 patients progress to end-stage renal disease, but its pathogenesis is unclear.

**Methods:**

This study enrolled 12 IgAN-S0 (without segmental sclerosis) and 19 IgAN-S1 (with segmental sclerosis) patients, performed 16S rRNA gene sequencing on fecal samples, and analyzed gut microbiota composition and functions.

**Results:**

S1 had enriched Firmicutes and Patescibacteria while S0 had more Proteobacteria, Campylobacterota, and Desulfobacterota; LEfSe analysis identified Subdoligranulum and unclassified_Erysipelotrichaceae_UCG-003 as S1-specific biomarkers and Phascolarctobacterium, Streptococcus_parasanguinis, and Proteobacteria as S0 biomarkers (P<0.05). Functional prediction showed S1 was enriched in pro-inflammatory pathways like endoplasmic reticulum stress and secondary bile acid biosynthesis, while S0 had activated protective pathways such as cytochrome P450 drug metabolism and ubiquitin system.

**Conclusions:**

This study reveals gut microbiota dysregulation is closely associated with IgAN segmental sclerosis, with S1 showing pro-inflammatory microbial profiles and S0 retaining protective functions, providing new insights into gut-kidney axis mechanisms and potential microbiome-targeted therapies for IgAN.

## 1 Introduction

IgA nephropathy (IgAN) is a prevalent form of immune - complex - mediated glomerulonephritis. It is characterized by the deposition of IgA or IgA - dominated immune complexes in the mesangial region of the glomerulus, accompanied by mesangial cell proliferation and an increase in mesangial stroma (Wang et al., 2020). Globally, the overall incidence of IgA nephropathy is at least 2.5 cases per 100,000 population (Pattrapornpisut et al., 2021). Regrettably, approximately 20% - 40% of IgAN patients progress to end - stage renal disease within 10 - 20 years after the initial renal biopsy (Wu et al., 2021). Focal segmental glomerulosclerosis (FSGS), a morphological marker of glomerular injury, is commonly observed in IgAN, being present in 70% of biopsy tissues (Bellur et al., 2024).

The Oxford classification has been instrumental in stratifying disease risk, among which the presence of segmental glomerulosclerosis (S1 lesion) stands out as a key independent predictor of poor renal outcome (Figueiredo et al., 2025). Studies such as the VALIGA cohort have consistently shown that S1 lesions are associated with heavier proteinuria, faster decline in glomerular filtration rate, and significantly lower renal survival (Bellur et al., 2024). While the prognostic importance of S1 lesions is well established (Haas, 2024), the underlying pathogenic drivers—particularly those extending beyond the glomerulus—remain incompletely understood. While immune complex deposition, mesangial cell dysfunction, and other established factors contribute to disease initiation, the specific mechanisms driving sclerosis formation and their interrelationships are not fully elucidated (Chiu et al., 2021). Key pathways under investigation include podocyte injury, immune-mediated processes such as complement activation, and hemodynamic stress. However, how these elements interact within the context of IgAN-S to accelerate disease progression continues to be an active area of inquiry (Chen et al., 2016; Trimarchi and Coppo, 2020; Chiu et al., 2021).

Studies have indicated that patients with IgA nephropathy exhibit an elevated abundance of Escherichia coli compared to healthy controls, while those with impaired renal function show alterations in Enterococcaceae, Moraxella, and Acinetobacter—changes primarily mediated through microbial involvement in bile acid metabolism pathways (Deng et al., 2024).Further evidence suggests that genera such as Akkermansia, Lachnospiraceae NK4A136 group, Lactococcus, and Butyricimonas are associated with renal function decline, metabolic disturbances, oxidative stress, and mitophagy dysfunction (Liu et al., 2024).Most recently, the taxonomic lineage Lactobacillus johnsonii has been identified as correlating with disease progression in chronic kidney disease, with its abundance closely linked to key clinical renal biomarkers (Miao et al., 2024).

Given the unresolved pathogenesis of IgAN-S and growing evidence implicating the gut microbiota in various diseases, this study aims to investigate the development of IgAN-S from a microbiome perspective. We compared the gut microbiota composition and functional profiles between patients with sclerotic lesions (S1) and those without (S0). By identifying microbial and metabolic differences between these groups, we seek to uncover potential microbiota-associated factors that may contribute to sclerotic progression. This approach may offer novel insights into the pathogenesis of IgAN-S and inform future targeted therapeutic strategies.

## 2 Method

### 2.1 Study design

In this study, a prospective clinical cohort study was conducted to analyze the gut microbiota in the feces of IgA nephropathy patients without segmental sclerosis, and IgA nephropathy patients with segmental sclerosis were used as controls. This study started in December 2022 and lasted for 2 years. All fecal samples in this study were from the participants in Zhejiang, China.

### 2.2 Study participants

**Figure 1:**
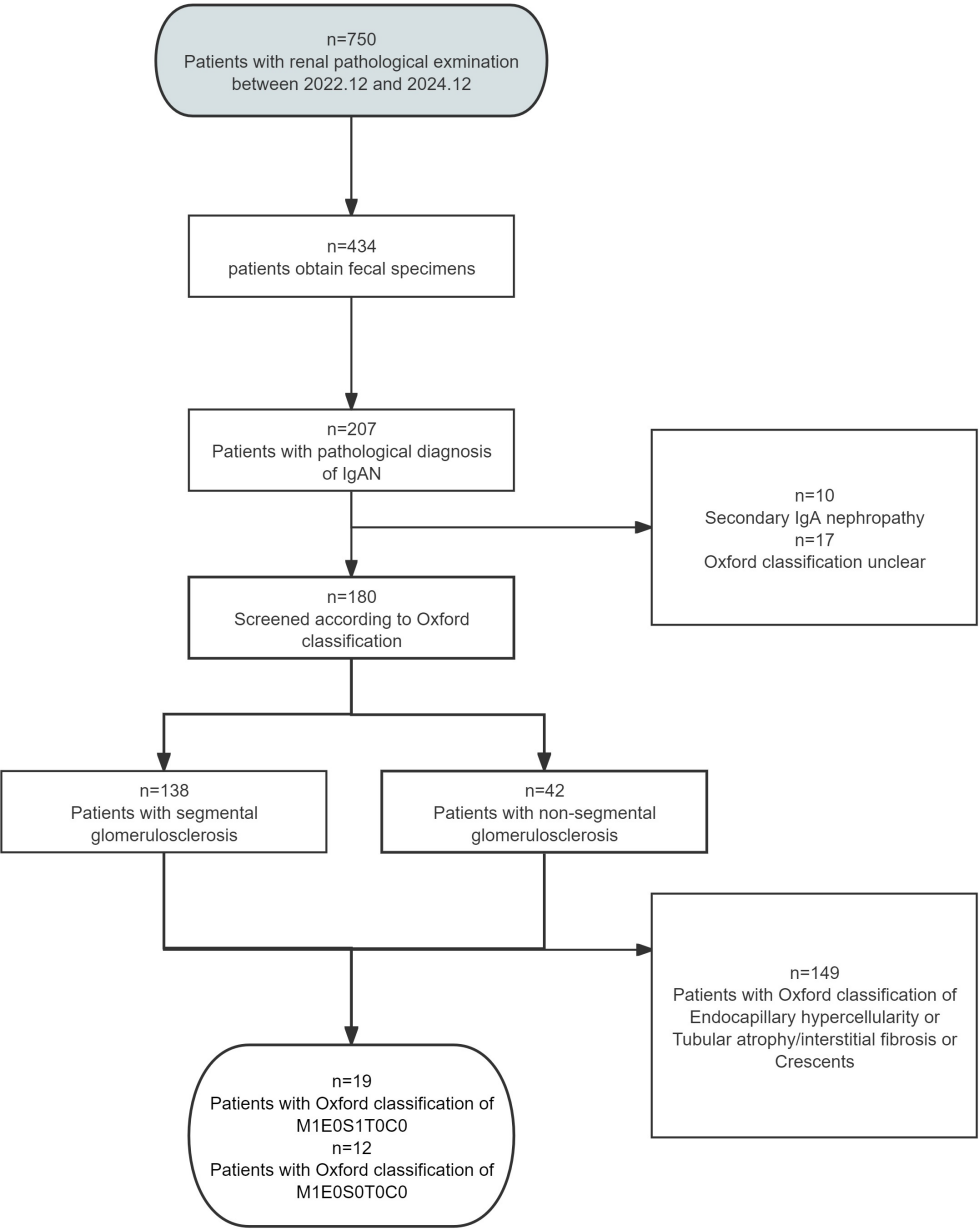
Flow chart of patients selection.

At the beginning of the study, fecal samples were collected from 750 patients who underwent renal biopsy in our hospital. A total of 434 samples were obtained, of which 207 samples were diagnosed as IgAN. Excluding the secondary IgAN and the unclear part of the Oxford classification, the remaining 180 patients were further screened according to the Oxford classification. Twelve patients with pathological diagnosis of IgA nephropathy and Oxford classification of M1E0S0T0C0 and 19 patients with Oxford classification of M1E0S1T0C0 were selected to study the characteristics of intestinal microbial composition in the two groups (**Figure 1**).

Diagnostic standard were as follows:

Pathological diagnostic criteria of IgA nephropathy:

Immunofluorescence showed granular and mass-like deposition of IgA or IgA deposition in the mesangial area, with or without other immunoglobulins (Kidney Disease: Improving Global Outcomes (KDIGO) Glomerular Diseases Work Group, 2021).

Oxford classification diagnostic criteria:

M refers to mesangial cell proliferation, which is divided into M0 (no or mild mesangial cell proliferation) and M1 (moderate or severe mesangial cell proliferation) according to the degree of mesangial cell proliferation. E refers to the proliferation of capillary endothelial cells, E0 means no, E1 means yes; s refers to segmental sclerosis or adhesion, S0 is none, S1 is yes; t refers to renal tubular atrophy or renal interstitial fibrosis, T0 represents renal tubular atrophy or renal interstitial fibrosis ≤ 25%, T1 represents 26% -50%, T2 represents > 50%; c represents cell or cell fibrous crescent, C0 is absent, and C1 is present (Trimarchi et al., 2017).

The inclusion criteria were as follows:

1. Meet the pathological diagnostic criteria for IgA nephropathy;
2. Age 18-60 years old;
3. Patients who were conscious, had no language communication barriers; gave informed consent and signed the Patient Informed Consent Form.

The exclusion criteria were as follows:

1. The pathological results of renal biopsy lack the Oxford classification;
2. Combined with Henoch-Schonlein purpura nephritis, lupus nephritis, alcoholic cirrhosis, rheumatoid arthritis, ankylosing spondylitis and other diseases that can cause secondary glomerular mesangial IgA deposition;
3. Those who have used antibiotics, prebiotics, probiotics or proton pump inhibitors in the past 1 month and other drugs that may affect the intestinal microbiota;
4. Those who fail to keep stool specimens as required or withdraw from the study for other reasons.

### 2.3 Fecal sample collection and storage

A fresh fecal sample is taken through a rectal swab. The patient is instructed to thoroughly clean the anal area with water and 75% alcohol before collection, scrape the fecal sample expelled from the anus using a sterile swab, place it in a sterile tube, and store it immediately in a -80°C freezer.

### 2.4 DNA extraction

Total genomic DNA of microbial communities was extracted from fecal samples following the instructions using the FastPure Stool DNA Isolation Kit. Subsequently, the integrity of the extracted genomic DNA was checked by 1% agarose gel electrophoresis, and the concentration and purity of the DNA were determined using a NanoDrop2000 spectrophotometer.

### 2.5 PCR amplification and sequencing library construction

Using the extracted DNA as a template, specific primers carrying Barcode sequences, 338F (5 ‘-ACTCCTACGGGAGGCAGCAG-3’) and 806R (5 ‘-GGACTACHVGGGTWTCTAAT-3’), were used to perform PCR amplification of the V3-V4 variable region of the 16S rRNA gene. The PCR products from the same sample were mixed and then purified using 2% agarose gel electrophoresis to recover the target fragment. The size of the recovered fragment was determined by 2% agarose gel electrophoresis, and the purified product was quantified using Synergy HTX. The purified PCR products were indexed using NEXTFLEX Rapid DNA-Seq Kit and sequenced on the NextSeq 2000 PE300 platform provided by Illumina.

### 2.6 16SrRNA sequencing and analysis

**Figure 2:**
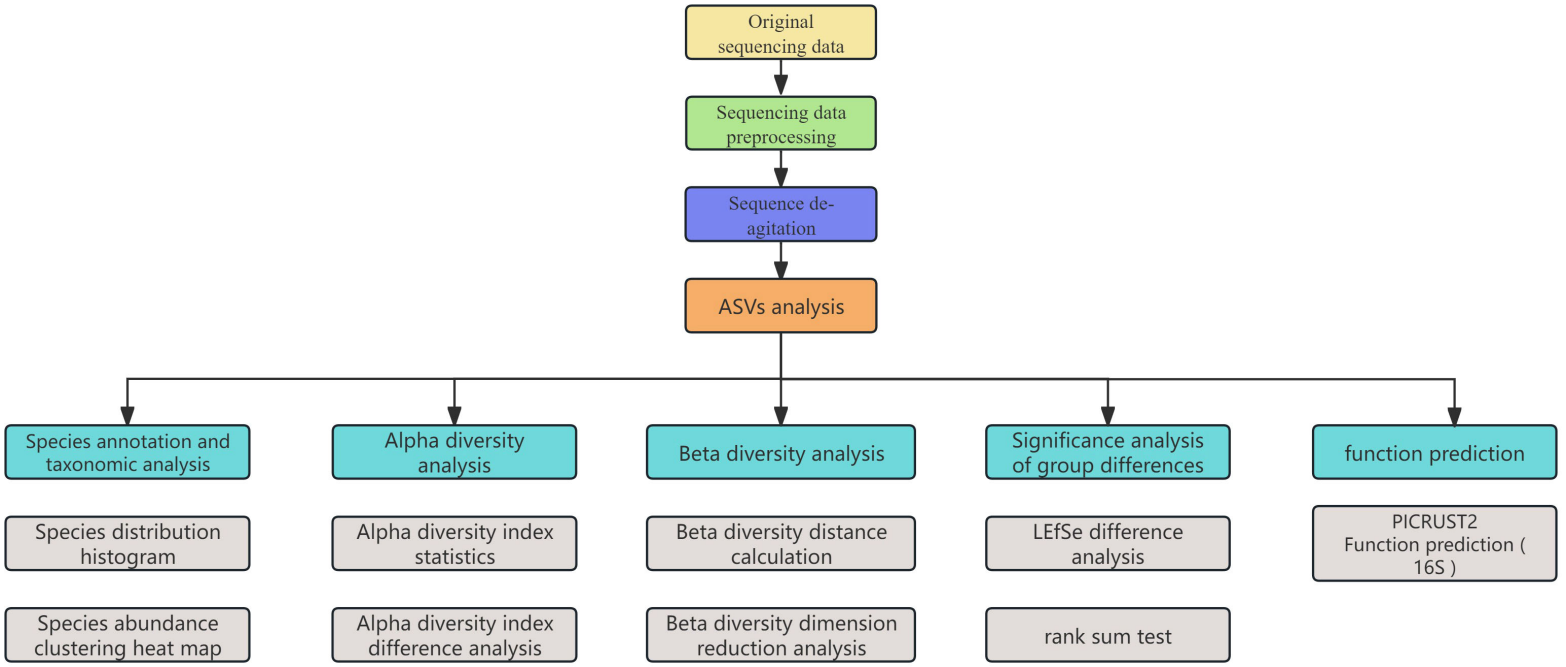
Statistical analysis flow chart.

Fastp software was used to control the quality of the double-ended original sequencing sequence, and FLASH software was used for splicing. Used the cutadapt software to identify and remove the primer sequence to obtain Clean Reads; the DADA2 method in QIIME2 software was used to denoise the sequence to obtain amplicon sequence variants (ASVs), Merged Reads and remove the chimera sequence to obtain the final valid data (Non-chimeric Reads). The Silva 16SrRNA gene database (v138) was annotated by RDP classifier, and the confidence threshold was 70%, and the community composition of each sample was counted at different species classification levels. PICRUSt2 (version2.2.0) software was used for 16S function prediction analysis. Alpha diversity index was calculated by mothur software, and the difference of Alpha diversity between groups was statistically analyzed by Wilcoxon rank sum test. Principal component analysis based on Bray-Curtis distance algorithm was used to evaluate the similarity of microbial community structure between samples, and non-parametric test was used to analyze the significant differences of microbial community structure between groups. In addition, LEfSe (Line Discriminant Analysis Effect Size) analysis was used to identify bacterial groups with significant differences in abundance between groups from phylum to species level. In the statistical analysis, P value less than 0.05 was considered statistically significant (**Figure 2**).

## 3 Results

### 3.1 General characteristics of all participants

In the comparison of general clinical data, S0 and S1 were statistically different in age (p=0.004) and not statistically different in terms of gender and course (p>0.05). In the comparison of test result, S0 and S1 did not show statistical difference in leucocyte, hemoglobin, thrombocyte, prothrombin time, thrombin time, d-dimer assay, fibrinogen, serum creatinine, blood albumin, urine red blood cell, 24-hour urine protein and glomerular filtration rate (p>0.05) (**Table 1**).

**Table 1:** General characteristics of participants.

Characteristic	S0 (n=12)	S1 (n=19)	p
Age (year)	48.75 ± 11.12	34.23 ± 11.42	0.004
Sex (male/female)	7/5	8/11	0.379
Course of disease (mouth)	18.38 ± 24.35	38.11 ± 38.04	0.123
Leucocyte (×10^9^/L)	6.35 ± 1.65	6.35 ± 1.49	0.992
Hemoglobin (g/L)	131.00 ± 16.87	136.78 ± 16.99	0.368
Thrombocyte (×10^9^/L)	222.33 ± 45.72	230.94 ± 54.92	0.657
Prothrombin time (s)	10.81 ± 0.53	10.90 ± 0.71	0.705
Thrombin time (s)	16.77 ± 0.84	16.44 ± 3.85	0.778
D-dimer assay (mg/L)	0.51 ± 0.64	0.31 ± 0.32	0.281
Fibrinogen (mg/dL)	3.31 ± 1.62	2.94 ± 0.40	0.371
Serum creatinine (μmol/L)	108.64 ± 112.40	84.44 ± 26.69	0.384
Blood albumin (g/L)	37.66 ± 4.43	38.60 ± 5.06	0.605
Urine red blood cell (/HPF)	26.67 ± 21.51	34.67 ± 37.65	0.512
24-hour urine protein (g/24h)	0.70 ± 0.59	1.18 ± 0.71	0.063
Glomerular filtration rate (mL/min/1.73m²)	82.79 ± 31.40	83.58 ± 21.69	0.935

### 3.2 ASVs analysis

**Figure 3:**
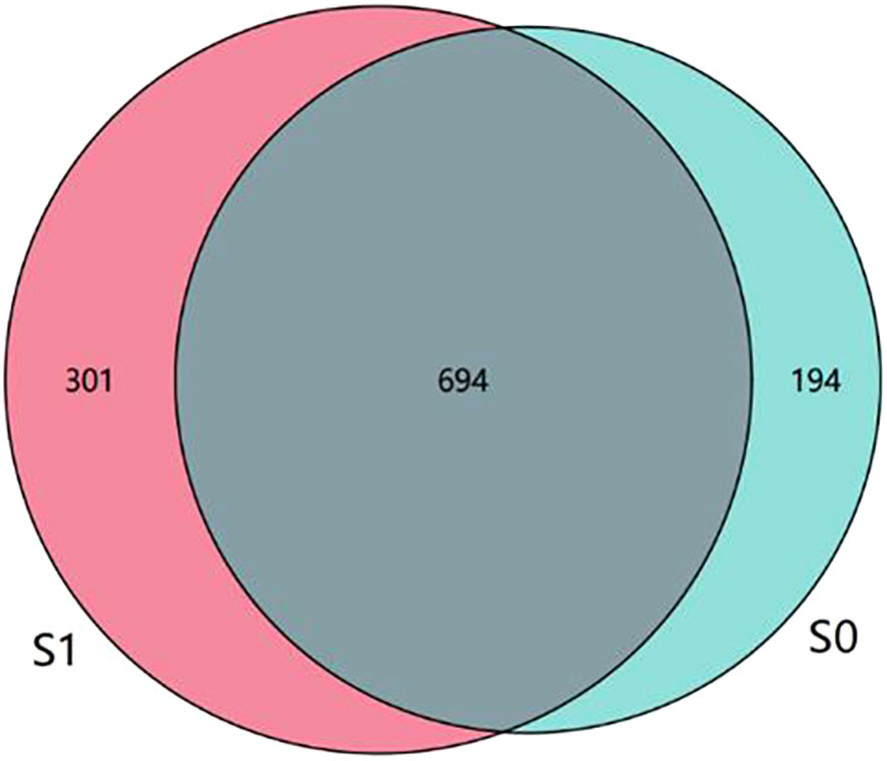
The intestinal flora Wayne diagram of S1 group and S0 group. There were 694 common ASVs in S1 group and S0 group, 301 individual ASVs in S1 group and 194 individual ASVs in S0 group.

Based on the concept clustering of ASVs, the results showed that there were 995 ASVs in S1 group and 888 ASVs in S0 group, and the ASVs in S1 group were more than those in S0 group. According to the ASV Wayne diagram, it can be seen that there are 694 ASVs shared by S1 group and S0 group, 301 ASVs owned by S1 group alone, and 194 ASVs owned by S0 group alone. This preliminary indicates that there are some differences in the types and composition of intestinal microorganisms between the two groups (**Figure 3**).

### 3.3 Species diversity analysis

#### 3.3.1 Alpha diversity analysis

**Figure 4:**
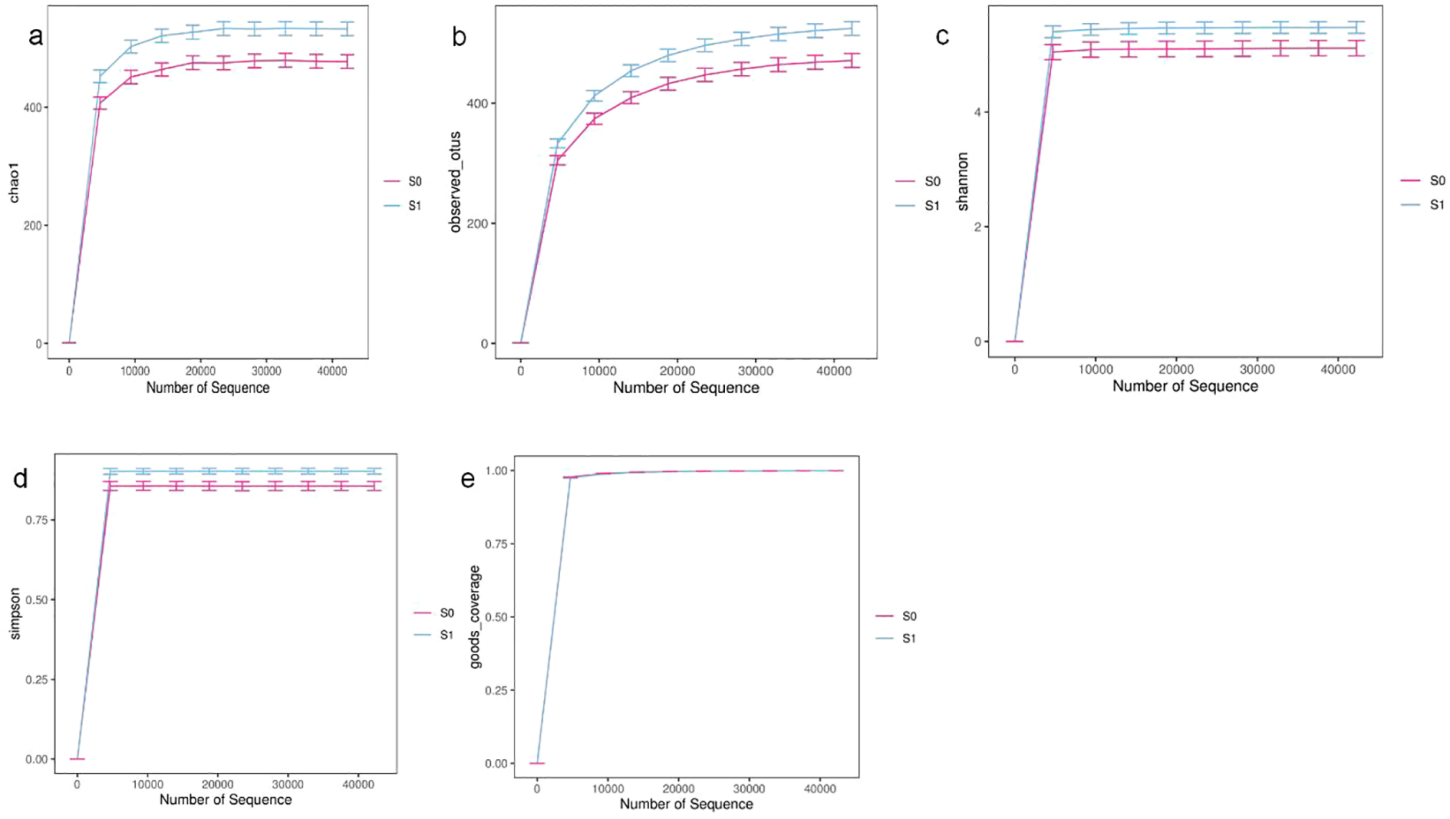
Alpha diversity analysis dilution curve chart. **(a)** Chao 1 index; **(b)** Observed species index; **(c)** Shannon index; **(d)** Simpson index; **(e)** Goods coverage index. The different colors in the figure represent the samples of different groups, the abscissa represents the number of randomly selected sequences, and the ordinate represents the index value.

According to the Alpha diversity index, the dilution curve was drawn. Combined with the Chao1 index and the Observed species index, it can be seen that the number of intestinal microbial species in the S1 group is more than that in the S0 group. Combined with Shannon index and Simpson index, it can be seen that the intestinal microbial diversity and uniformity of S1 group are higher than those of S0 group. In addition, the Goods coverage index of the two groups was close to 1, and the dilution curve of each index tended to be gentle with the increase of sequencing amount, indicating that the sequencing amount and sequencing depth were sufficient, and the sequencing results could reflect the real situation of the sample microorganisms (**Figure 4**).

#### 3.3.2 Beta diversity analysis

**Figure 5:**
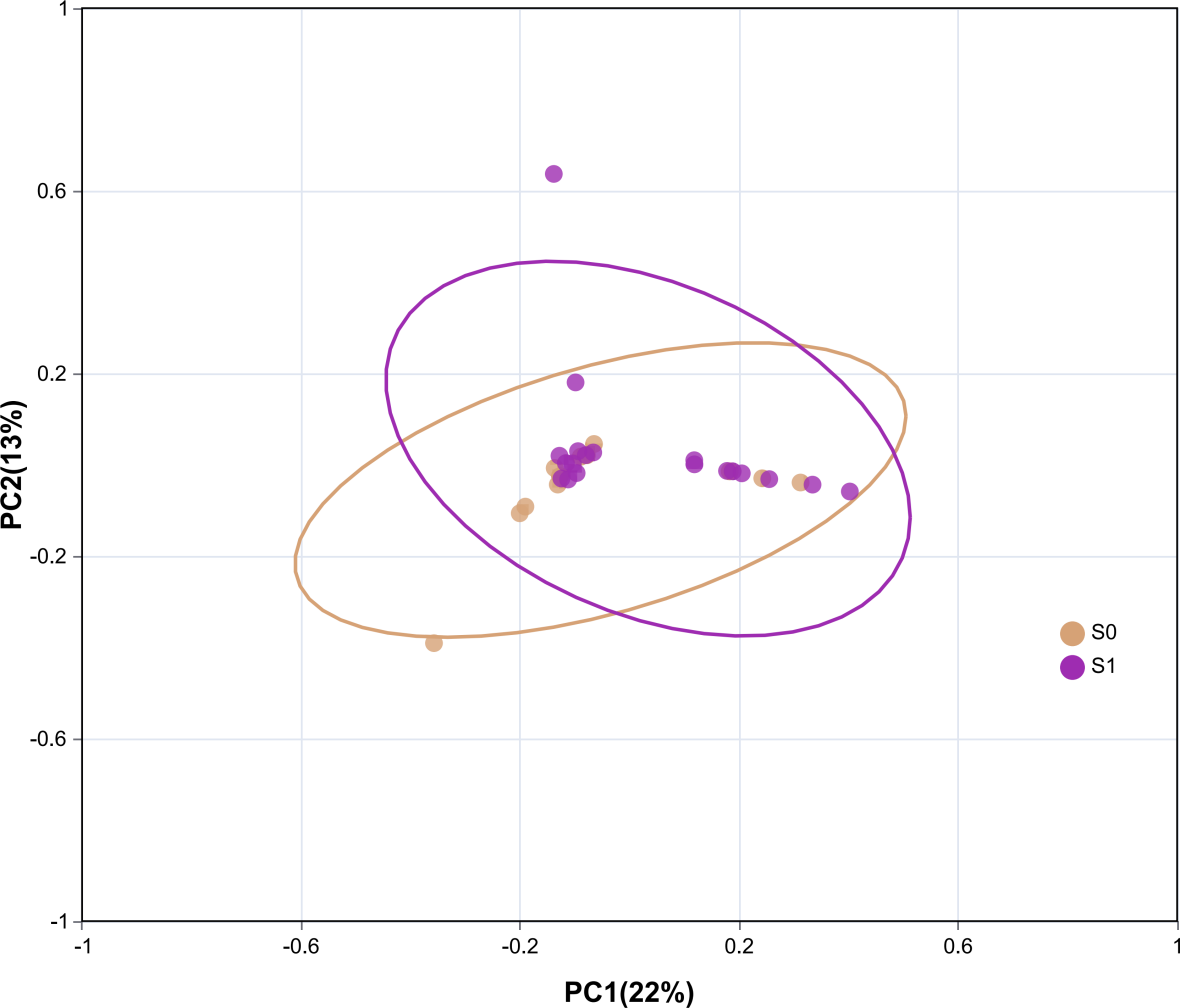
Two-dimensional coordinate diagram of Beta diversity analysis. Each point in the figure represents a sample; the oval circle is its 95% confidence interval. The abscissa represents the contribution value of the first principal component to the sample difference; the ordinate represents the contribution value of the second principal component to the sample difference.

By analyzing the PCA two-dimensional coordinate diagram, it can be seen that there is a certain separation trend between the S1 group and the S0 group, but the distance is small, suggesting that the species diversity of the intestinal flora of the two groups is similar (**Figure 5**).

### 3.4 Species difference analysis

#### 3.4.1 Species composition heat map

**Figure 6:**
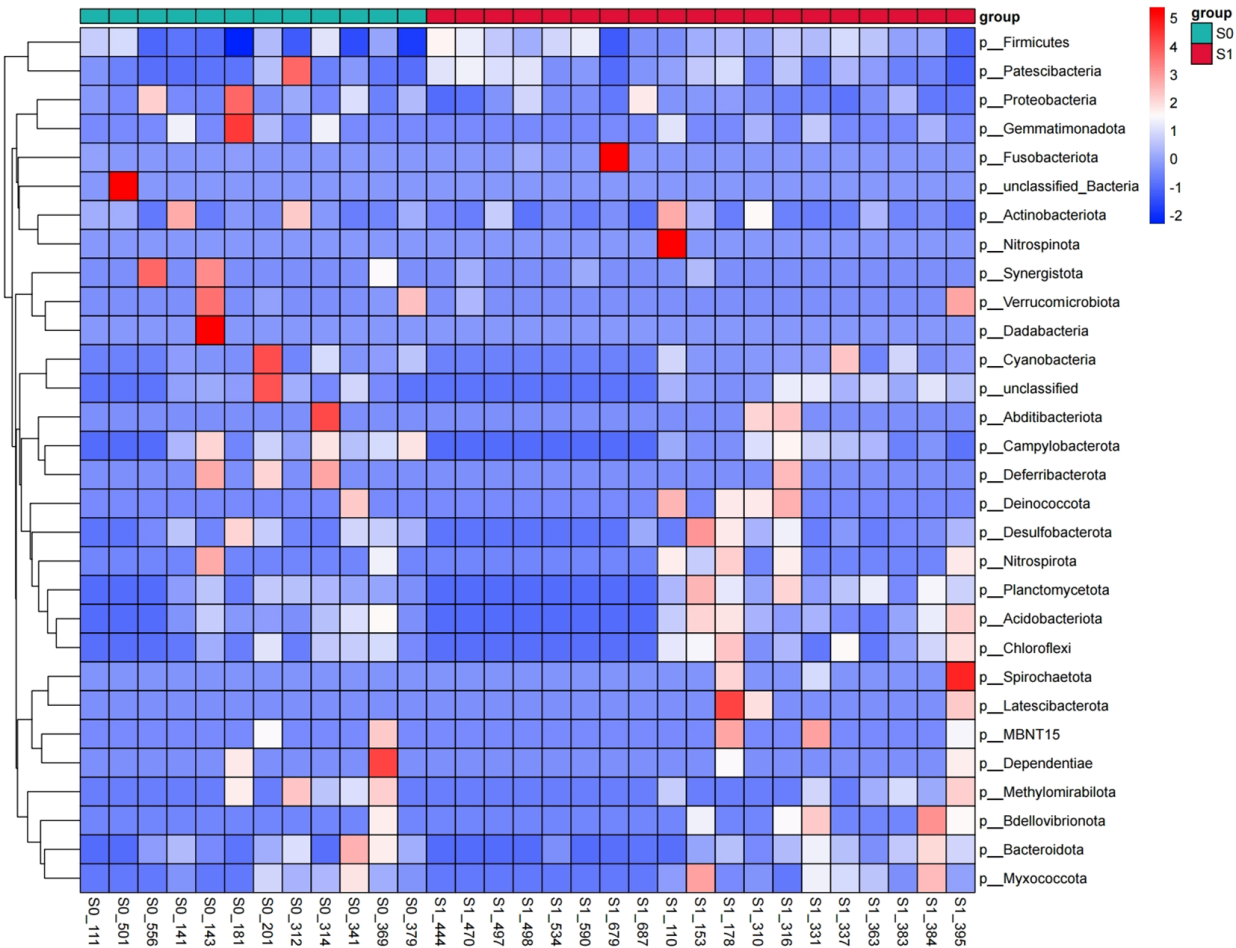
Species composition heat map. The transverse axis is different samples, and different colors represent different groups; the longitudinal axis is different intestinal microorganisms, different colors represent different abundances, and the relative proportion of a species from low to high among different samples is indicated by blue to red.

The cluster heat map of TOP30 species abundance at the phylum level showed that the overall community structure of intestinal microorganisms in S0 group and S1 group was similar, and there was no significant difference, but there was a significant difference in the abundance of individual phylum (**Figure 6**).

#### 3.4.2 Comparison of species abundance

**Figure 7:**
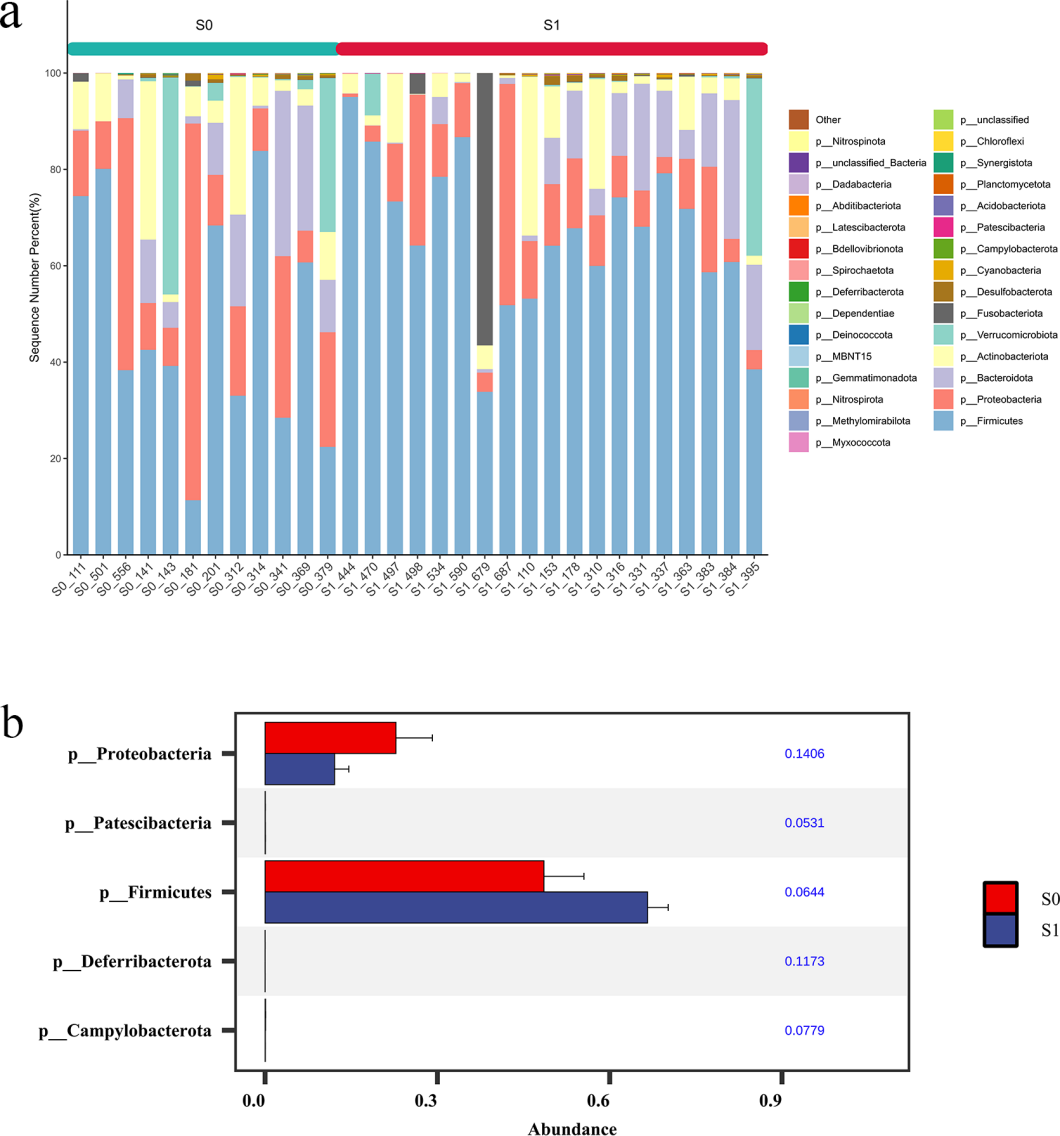
Comparison of species abundance at the phylum level. **(a)** TOP30 species abundance histogram at phylum level. The abscissa is the sample name; the ordinate is the relative abundance percentage. The stacking columns of different colors were the top 30 species with the largest relative abundance in each sample, and the remaining species were merged in ‘Others ‘. **(b)** Phylum level significant difference species abundance histogram.

At the Phylum level. The top 10 dominant phyla in S1 group and S0 group were Firmicutes, Proteobacteria, Bacteroidota, Actinobacteriota, Verrucomicrobiota, Fusobacteriota, Desulfobacterota, Cyanobacteria, Campylobacterota and Patescibacteria. The Wilcoxon rank sum test was used to analyze the species differences between the two groups. The results showed that the abundance of Firmicutes and Patescibacteria in the S1 group was significantly higher than that in the S0 group (p < 0.05), and the abundance of Proteobacteria, Campylobacterota and Desulfobacterota in the S0 group was significantly higher than that in the S1 group (p < 0.05) (**Figure 7**).

**Figure 8:**
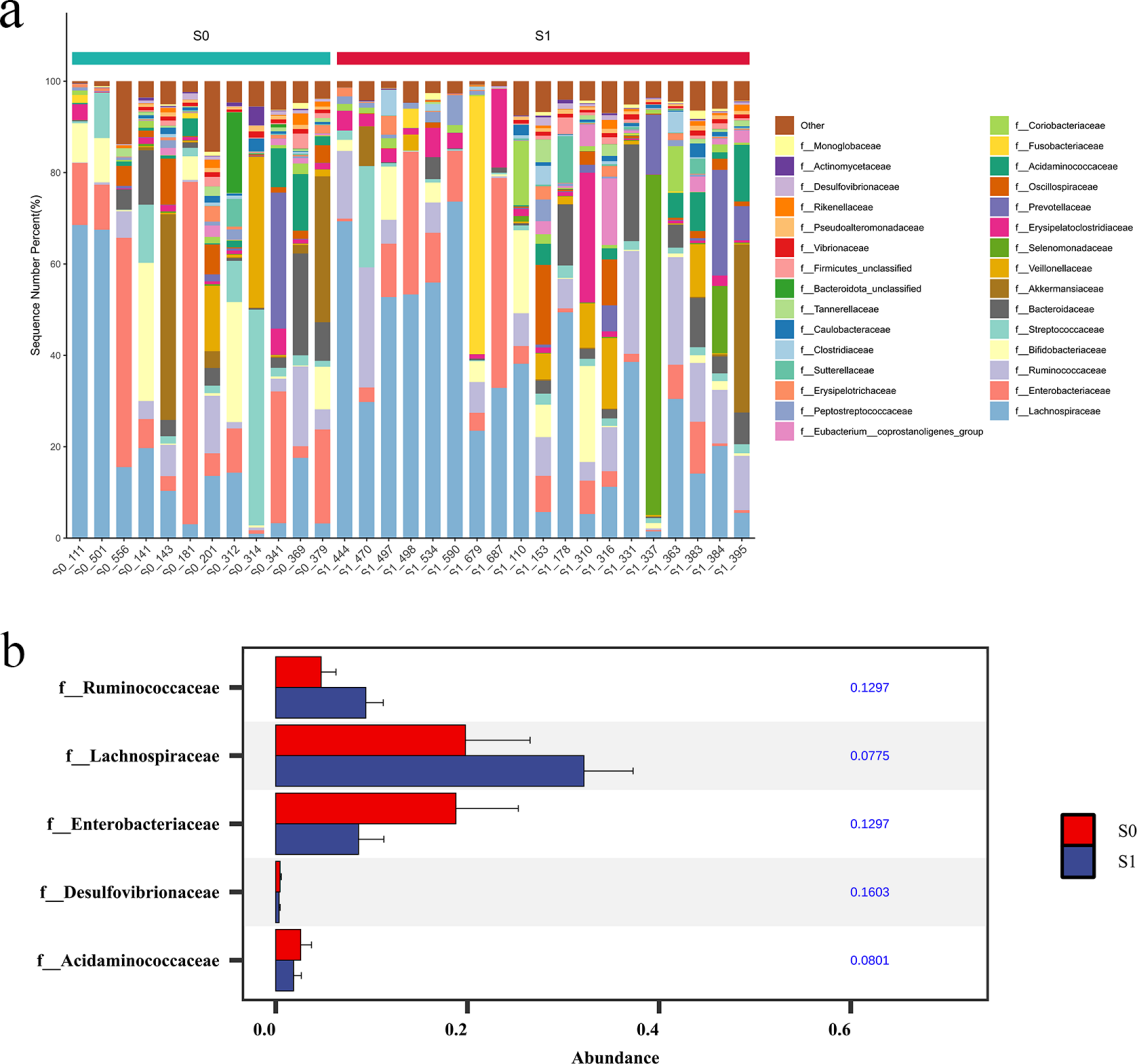
Comparison of species abundance at the family level. **(a)** Histogram of TOP30 species abundance at family level. The abscissa is the sample name; the ordinate is the relative abundance percentage. The stacked columns of different colors were the species with the largest relative abundance ranking 30 in each sample, and the remaining species were merged in ‘Others ‘. **(b)** Column diagram of species abundance with significant difference at family level.

At the Family level. The top 10 dominant families in S1 group and S0 group were Lachnospiraceae, Enterobacteriaceae, Ruminococcaceae, Bifidobacteriaceae, Streptococcaceae, Bacteroidaceae, Akkermansiaceae, Veillonellaceae, Selenomonadaceae, Erysipelatoclostridiaceae. Further Wilcoxon rank sum test was used to analyze the species differences between the two groups. The results showed that the abundance of Ruminococcaceae and Lachnospiraceae in the S1 group was significantly higher than that in the S0 group (p < 0.05), and the abundance of Enterobacteriaceae and Desulfovibriaceae in the S0 group was significantly higher than that in the S0 group (p < 0.05) (**Figure 8**).

**Figure 9:**
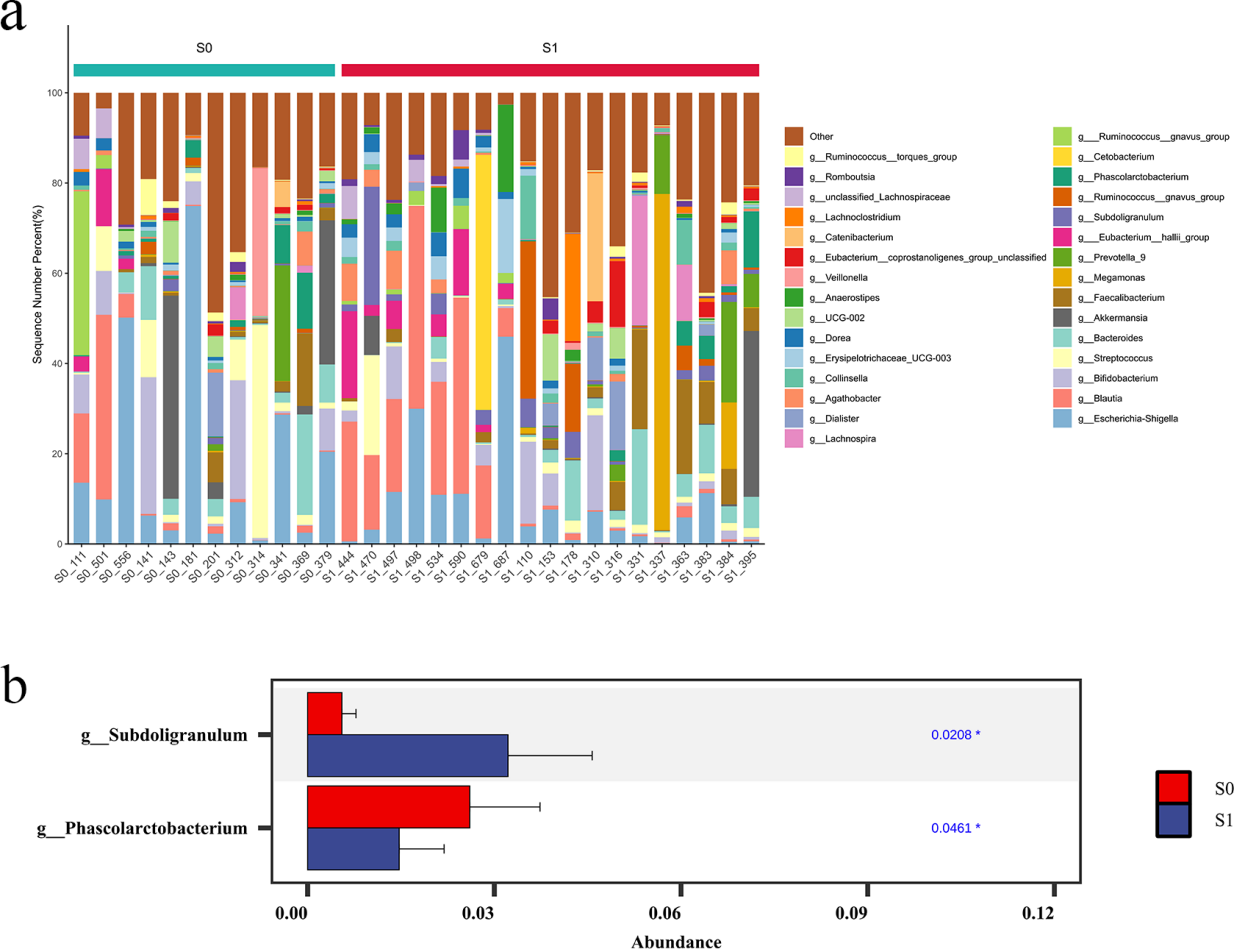
Comparison of species abundance at the genus level. **(a)** The species abundance histogram of TOP30 at genus level. The abscissa is the sample name; the ordinate is the relative abundance percentage. The stacked columns of different colors were the species with the largest relative abundance ranking 30 in each sample, and the remaining species were merged in ‘Others ‘. **(b)** Column diagram of species abundance with significant difference at genus level.

At the genus level. The top 10 dominant genera in S1 group and S0 group were Escherichia-Shigella, Blautia, Bifidobacterium, Streptococcus, Bacteroides, Akkermansia, Faecalibacterium, Megamonas, Prevotella_9 and Eubacterium:hallii_group. The species difference between the two groups was analyzed by Wilcoxon rank sum test. The results showed that the abundance of Subdoligranulum in S1 group was significantly higher than that in S0 group (p < 0.05), and the abundance of Phascolarctobacterium in S0 group was significantly higher than that in S1 group (p < 0.05) (**Figure 9**).

**Figure 10:**
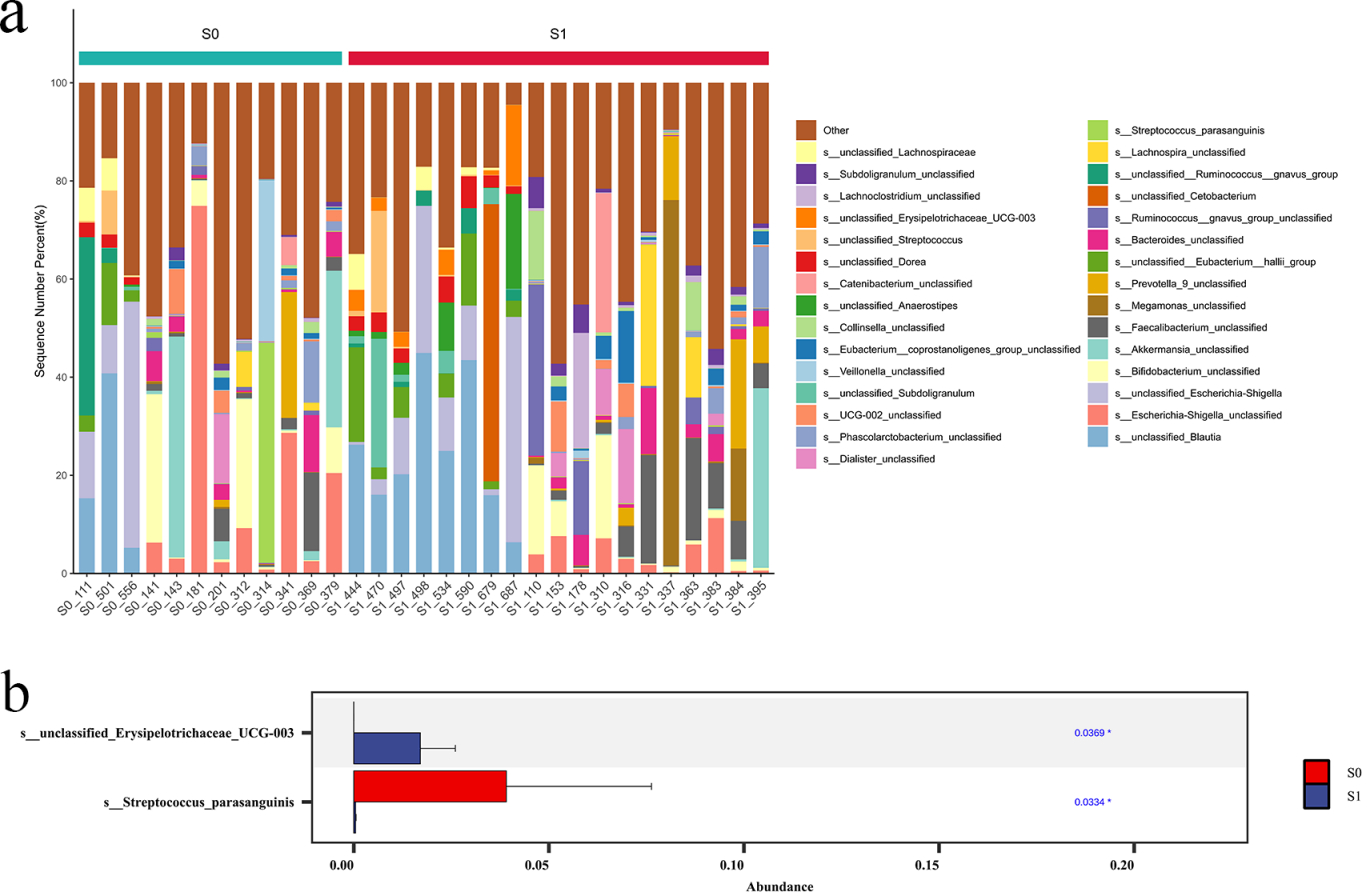
Comparison of species abundance at the species level. **(a)** Species-level TOP30 species abundance histogram. The abscissa is the sample name; the ordinate is the relative abundance percentage. The stacked columns of different colors were the species with the largest relative abundance ranking 30 in each sample, and the remaining species were merged in ‘Others ‘. **(b)** Species abundance histogram with significant difference at species level.

At the species level, the top 10 dominant species in S1 group and S0 group were unclassified _ Blautia, Escherichia-Shigella_unclassified, unclassified_Escherichia-Shigella, Bifidobacterium_unclassified, Akkermansia_unclassified, Faecalibacterium_unclassified, Megamonas_unclassified, Prevotella_9_unclassified, unclassified:Eubacterium:hallii_group, Bacteroides_unclassified. Further Wilcoxon rank sum test was used to analyze the species difference between the two groups. The results showed that the abundance of unclassified _ Erysipelotrichaceae_UCG-003 in S1 group was significantly higher than that in S0 group (p < 0.05), and the abundance of Streptococcus_parasanguinis in S0 group was significantly higher than that in S1 group (P < 0.05) (**Figure 10**).

### 3.4.3 LEfSe analysis

**Figure 11:**
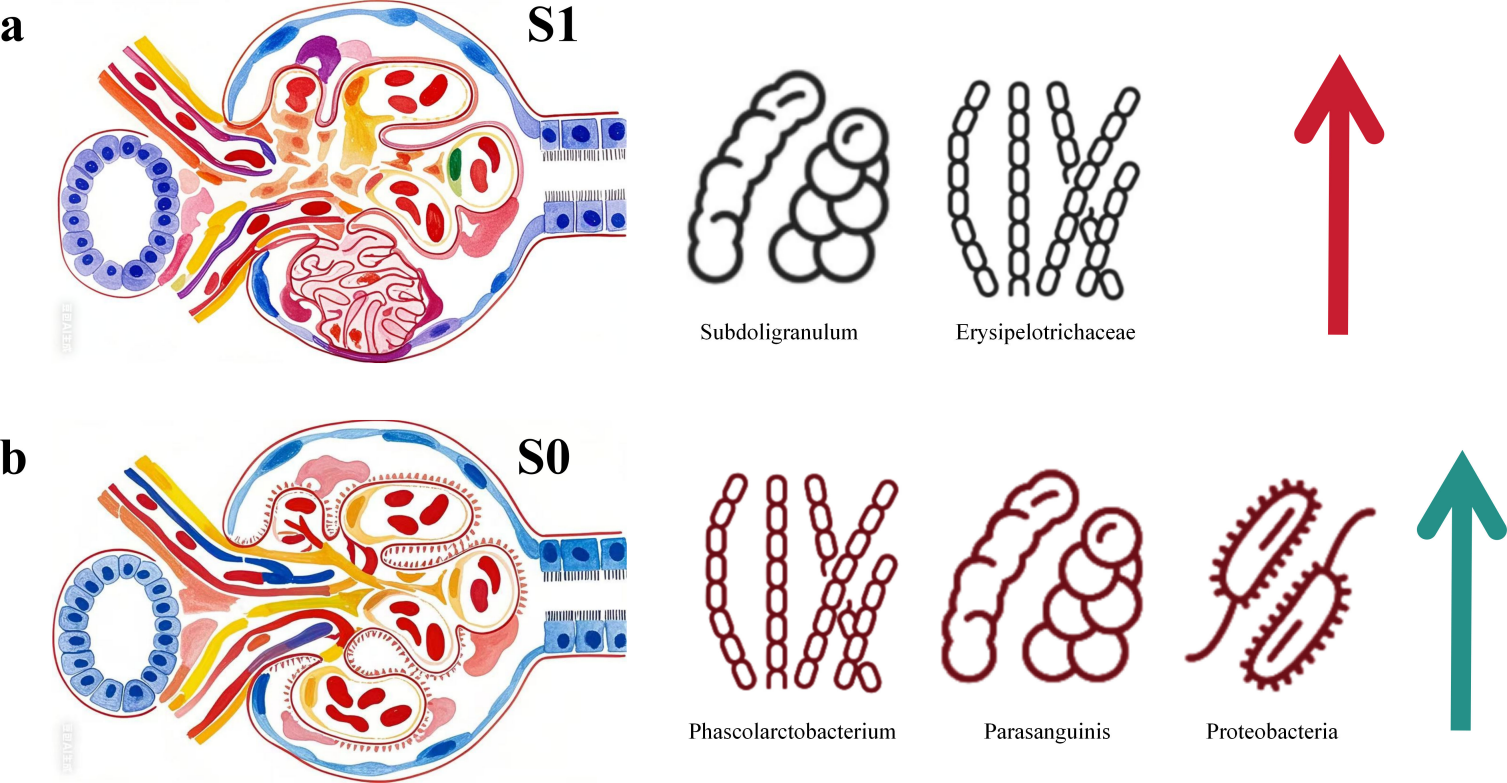
LEfSe analysis. **(A)** Subdoligranulum and unclassified _ Erysipelotrichaceae _ UCG-003 were the dominant bacteria in the S1 group, and Phascolarctobacterium, Streptococcus _ parasanguinis and Proteobacteria were the dominant bacteria in the S0 group. **(B)** The difference screening threshold of LEfSe analysis: LDA value > 1.0, test P value < 0.05.

In this study, LEfSe analysis, combined with Wilcoxon rank sum test and LDA (Linear discriminant analysis) effect size, was used to find intestinal biomarkers with statistical differences between S1 group and S0 group. The LDA histogram showed that there were five different species between the S1 group and S0 group. Among them, there were two dominant bacteria in the S1 group, which were Subdoligranulum and unclassified_Erysipelotrichaceae_UCG-003, suggesting that these two bacteria have the potential to be used as intestinal microbial markers for segmental glomerulosclerosis. There were three dominant bacteria in the S0 group, namely Phascolarctobacterium, Streptococcus_parasanguinis, and Proteobacteria, suggesting that these three bacteria have the potential to be used as intestinal microbial markers for non-segmental glomerulosclerosis (**Figure 11**).

### 3.5 Predictive functional analysis

Kyoto Encyclopedia of Genes and Genomes (KEGG) is one of the databases commonly used in pathway research. KEGG describes a series of metabolic pathways and the relationship between metabolic pathways, covering information such as metabolism, genetic information, environmental information, and cellular processes. In this study, Phylogenetic Investigation of Communities by Reconstruction of Unobserved States (PICRUSt2) software was used to compare the 16SrRNA characteristic sequence with the KEGG microbial functional genome database, and the gene information of unknown species was predicted according to the gene type and abundance information of known species, so as to predict the pathway of intestinal microbial community in the two groups.

**Figure 12:**
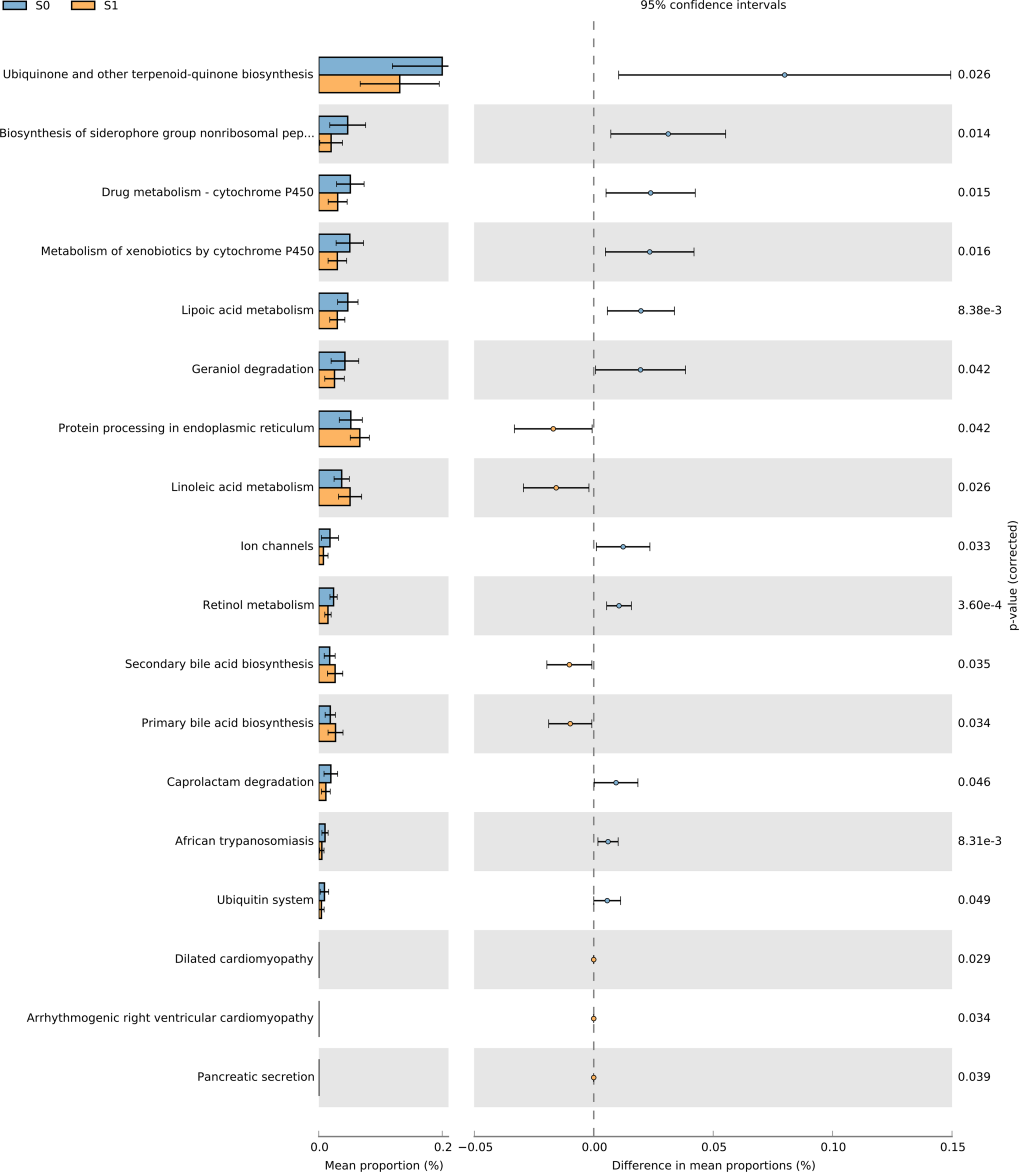
Predictive functional analysis.

The results of PICRUSt2 function prediction showed that the two groups of samples showed significant differences in 18 functional pathways. Among them, the S1 group samples were significantly enriched in 7 functional pathways (P < 0.05), including Protein processing in endoplasmic reticulum, Linoleic acid metabolism, Secondary bile acid biosynthesis, Primary bile acid biosynthesis, Dilated cardiomyopathy, Arrhythmogenic right ventricular cardiomyopathy, and Pancreatic secretion. The S0 group samples were significantly enriched in 11 functional pathways (P < 0.05), including Ubiquinone and other terpenoid-quinone biosynthesis, Biosynthesis of Siderophore Group Nonribosomal Peptides, Drug metabolism - cytochrome P450, Metabolism of xenobiotics by cytochrome P450, Lipoic acid metabolism, Geraniol degradation, Ion channels, Retinol metabolism, Caprolactam degradation, African trypanosomiasis, and Ubiquitin system (**Figure 12**).

## 4 Discussion

### 4.1 Importance of IgAN S1

In IgA nephropathy (IgAN), segmental glomerulosclerosis (S lesion) defined by the Oxford classification is a critical pathological feature and an independent risk factor for disease progression, closely associated with increased proteinuria, accelerated decline in glomerular filtration rate (GFR), and poor renal prognosis (Bellur et al., 2017). Subtypes of S lesions, such as tip lesions and podocyte hypertrophy, indicate active podocytopathy and are linked to higher baseline proteinuria and more rapid renal function deterioration, particularly in patients without immunosuppressive treatment (Bellur et al., 2017). The presence of S lesions also reflects complex pathological mechanisms, including podocyte injury, complement activation, and mesangial cell dysfunction, which collectively drive glomerular scarring and tubulointerstitial fibrosis (Xie et al., 2015; Chiu et al., 2021). Our study further contextualizes these findings by identifying distinct gut microbial signatures in S lesion-positive (S1) and S lesion-negative (S0) IgAN patients. Specifically, the S1 group was characterized by enriched Subdoligranulum and unclassified_Erysipelotrichaceae_UCG-003, which may exacerbate glomerular injury through pathways like endoplasmic reticulum stress and dysregulated bile acid metabolism (Bellur et al., 2017). In contrast, the S0 group harbored bacteria associated with xenobiotic metabolism and redox balance, potentially reflecting a less aggressive inflammatory milieu (Bellur et al., 2017). These results align with prior research demonstrating that S lesions in IgAN are not merely markers of chronicity but active drivers of disease progression, underscoring the importance of integrating pathological subclassification with microbiome profiling to refine risk stratification and therapeutic strategies in IgAN.

### 4.2 Mechanisms of IgAN S1 formation

The formation of segmental sclerosis in IgA nephropathy (IgAN) involves multiple interacting mechanisms. Podocyte injury is central, with gene mutations (e.g., NPHS2, TRPC6) disrupting the filtration barrier or calcium homeostasis to drive proteinuria and sclerotic progression (Singh et al., 2022). Soluble urokinase receptor (suPAR) exacerbates podocyte loss via integrin-mediated oxidative stress and Src kinase activation (Kim et al., 2018). Immune dysregulation plays a key role, including activation of complement pathways (elevated C3a, C5a, C5b-9) (Huang et al., 2020), anti-nephrin antibodies (Shirai et al., 2024), and B-cell abnormalities (e.g., increased memory B cells and plasmablasts) (Liu et al., 2021). In transplanted kidneys, metabolic/structural changes—such as podocyte overexpression of vascular endothelial growth factor A (VEGF-A) disrupting endothelial function and parietal epithelial cell dedifferentiation promoting collagen deposition—are critical (Gallon et al., 2025). Cytoskeletal proteins (e.g., Btg2 inducing podocyte-mesenchymal transition via the Smad3 pathway) (Dan Hu et al., 2023) and mitochondrial dysfunction (oxidative stress, dynamics defects) (Li et al., 2023) further propagate sclerosis. Genetic factors (e.g., APOL1 risk variants in African populations) (Bruno and Edwards, 2021), hemodynamic stress (obesity-related glomerular hyperfiltration) (Sandino et al., 2021), and cellular senescence (tubular p16INK4A upregulation) (Verzola et al., 2020) act as independent risk factors. Our study’s findings of distinct intestinal microbial markers between segmental sclerosis-positive (S1) and -negative (S0) groups suggest gut microbiota dysregulation may contribute to segmental sclerosis via the gut-kidney axis, potentially through metabolic or immune cross-reactivity pathways, offering new insights into its pathogenesis.

### 4.3 Gut microbiota changes and significance in IgAN S1 in our study

Unclassified_Erysipelotrichaceae_UCG-003, a member of the Erysipelotrichaceae family, is associated with detrimental effects in the S1 group (segmental glomerulosclerosis-positive IgAN). Erysipelotrichaceae dysbiosis leading to tryptophan metabolism imbalance promotes increased precursors such as indole and p-cresol, which are subsequently converted in the liver into protein-bound uremic toxins such as indoxyl sulfate (IXS) and p-cresyl sulfate (pCS) (Miao et al., 2025). These toxins, particularly their free forms, show a strong negative correlation with renal function (eGFR) and are reliable biomarkers for CKD progression (Corradi et al., 2024). They act as endogenous ligands activating signaling pathways such as AHR and NF-κB (Miao et al., 2025), promoting renal inflammation and fibrosis (He et al., 2025).

In the S1 group (IgAN with segmental glomerulosclerosis positive), Subdoligranulum demonstrates detrimental effects associated with metabolic inflammation and intestinal barrier dysfunction, which may further exacerbate the pathological progression in this subgroup. Changes in its abundance are linked to metabolic disturbances and may impair intestinal barrier integrity by reducing the production of short-chain fatty acids (SCFAs) (Kwiatkowska et al., 2024; Li et al., 2025). Therefore, the enrichment of Subdoligranulum and Erysipelotrichaceae UCG-003 in the S1 group may exacerbate the progression of IgA nephropathy through a synergistic and potentially additive mechanism involving damage to intestinal barrier function and increased bacterial endotoxin translocation (Cuartero et al., 2024; Li et al., 2024; Jin et al., 2026), which promotes the entry of uremic toxin precursors (such as indole and p-cresol) into systemic circulation and further activates signaling pathways including AHR, NF-κB, and PKC/PI3K (He et al., 2025).

In contrast, the S0 group (segmental glomerulosclerosis-negative IgAN) is enriched with potentially protective bacteria. Phascolarctobacterium is a known SCFA-producing bacterium, and its primary metabolite, propionate, belongs to the SCFA family (Li et al., 2025; Ni et al., 2025). Studies have indicated that SCFAs (e.g., propionate, butyrate) can inhibit the NF-κB pathway and enhance intestinal barrier integrity, thereby alleviating renal inflammation and fibrosis (He et al., 2025; Jin et al., 2026). Mendelian randomization studies support that the renal benefits of protective gut microbiota may be mediated by modulating the host’s blood metabolome (e.g., increasing levels of beneficial metabolites) (Cao et al., 2025). Additionally, research suggests that natural products (e.g., certain polysaccharides) can increase SCFA production by modulating the gut microbiota, further improving kidney disease (Zhang et al., 2022). Furthermore, Phascolarctobacterium interacts with Bacteroides thetaiotaomicron by utilizing succinate to generate propionate, promoting microbial symbiosis (Ikeyama et al., 2020; Ni et al., 2025).

Streptococcus_parasanguinis, a dominant bacterium in the S0 group, contributes to gut health via oral-gut axis interactions. As an oral commensal, it produces hydrogen peroxide to modulate microbial diversity and adheres to collagen, supporting mucosal integrity (Fultz et al., 2022; Li et al., 2025). In mouse pups, breast milk-derived S. parasanguinis upregulates anti-inflammatory Treg-related genes (e.g., Foxp3, TGF-β) in the ileum (Tsunoda et al., 2018; Fultz et al., 2022), a mechanism that may reduce glomerular immune complex deposition in IgAN. Its depletion in periodontitis patients correlates with mucosal barrier dysfunction (Chen et al., 2019; Fultz et al., 2022), highlighting its protective role.

The Proteobacteria phylum in the S0 group comprises species with diverse metabolic functions. Notably, certain members of this phylum (such as Escherichia coli) have been clearly demonstrated to metabolize tryptophan, producing key precursors like indole, which play a critical role in regulating immune balance and maintaining intestinal barrier function (Miao et al., 2025). Large-scale blood metabolome analyses indicate that specific gut microbiota are significantly associated with host circulatory levels of lipid metabolites and amino acid metabolites (Cao et al., 2025). Furthermore, this study found that specific Proteobacteria subgroups, including the model strain Escherichia coli str. K-12 substr. MG1655, are capable of elevating alpha-ketoglutarate (α-KG) levels (Cao et al., 2025). This metabolite exhibits anti-inflammatory and antioxidant properties, suggesting a potential inhibitory role in the progression of glomerulosclerosis. In this study, the Proteobacteria in the S0 group may include more beneficial species, which maintain ecological balance by promoting nutrient cycling and reducing the accumulation of toxic metabolites (Deng et al., 2021; Barron et al., 2024; Li et al., 2025).

### 4.4 Discoveries and significance of IgAN S1 microbiota functions in our study

Functional pathway analysis using PICRUSt2 revealed distinct molecular mechanisms differentiating the S1 (sclerotic) and S0 (non-sclerotic) phenotypes. The S1 group was characterized by enrichment in pathways promoting endoplasmic reticulum (ER) stress, such as “Protein processing in endoplasmic reticulum.” This involves mutant proteins (e.g., INF2, α-actinin-4) disrupting ER function, activating the unfolded protein response (UPR), and inducing podocyte apoptosis via caspase-12, supported by evidence of upregulated BIP and p-eIF2α in IgAN glomeruli (Lan et al., 2023; Tran et al., 2024). Dysregulated “Linoleic acid metabolism” in S1 promotes pro-inflammatory lipid peroxidation, generating reactive oxygen species that damage podocyte cytoskeletons (e.g., through α-actinin-4 oxidation) and link gut microbiota-driven inflammation to reduced SCFA production (Wu et al., 2022; Deng et al., 2024). Additionally, aberrant “Primary/secondary bile acid biosynthesis” in S1 exacerbates injury via FXR-TGR5 pathway activation and mitochondrial apoptosis induced by secondary bile acids (e.g., deoxycholic acid), with reduced Bifidobacterium abundance in IgAN patients connecting gut dysbiosis to bile acid toxicity (Widmeier et al., 2019; Deng et al., 2024).

In contrast, the S0 group maintains protective pathways including “Ubiquinone biosynthesis,” which preserves mitochondrial integrity via coenzyme Q10 and mitigates APOL1-associated ROS production (Wada et al., 2023; Mengarelli et al., 2025; Wang et al., 2025), and “Cytochrome P450 drug/xenobiotic metabolism,” which facilitates detoxification of nephrotoxic compounds (e.g., tacrolimus, bile acids) via enzymes such as CYP3A4 and CYP2C9 (Deng et al., 2022; Chebotareva et al., 2023). A robust “Ubiquitin system” in S0 ensures proteasomal clearance of misfolded proteins (e.g., nephrin, TRPC6), preventing aggregate formation and steroid resistance observed in S1, which is partly attributed to anti-UCH-L1 autoantibodies (Jia et al., 2025; Goeckeler-Fried et al., 2026).

Cross-pathway analysis underscores a fundamental contrast: S1 exhibits ER stress-overwhelmed proteostasis and pro-inflammatory lipid signaling, whereas S0 maintains mitochondrial-lipid metabolic balance and efficient gut-renal detoxification. This highlights the critical role of ER stress–ubiquitin–proteasome system crosstalk and microbiota–bile acid–CYP450 interactions in driving disease progression or conferring protection (Widmeier et al., 2019; Deng et al., 2024; Tran et al., 2024; Wang et al., 2025).

## 5 Conclusions

In conclusion, IgA nephropathy (IgAN) with segmental glomerulosclerosis (S lesion), particularly the S1 subtype, presents distinct pathological characteristics that are closely associated with gut microbiota composition. The S1 group exhibited an enrichment of potentially harmful bacteria, including Subdoligranulum and unclassified_Erysipelotrichaceae_UCG-003, whereas the S0 group was characterized by a higher abundance of beneficial bacteria such as Phascolarctobacterium. Functional pathway analysis further revealed that the S1 subtype is marked by activation of pathways promoting endoplasmic reticulum stress and inflammation, in contrast to the S0 subtype, which maintained protective metabolic and regulatory pathways. These findings underscore the importance of combining pathological subclassification with microbiome profiling in IgAN. Such an integrated approach may enhance risk stratification and could ultimately contribute to the development of microbiota-targeted therapies for improved disease management.

## 6 Limitations

This study has several limitations. First, the cross-sectional design precludes causal inference between the gut microbiome and pathological lesions. Second, the relatively small sample size may limit statistical power. Third, patients in the S1 group were younger than those in the S0 group in our cohort (p=0.004). It is noteworthy that prior studies have indicated that key pathological lesions in IgAN, such as glomerulosclerosis, exhibit a non-linear relationship with patient age, with the peak prevalence often observed in middle-aged rather than elderly groups (Zhang et al., 2023). This suggests that age per se is an intrinsic dimension and marker of disease heterogeneity (e.g., distinct clinicopathological phenotypes), rather than an independent confounder that can be simply adjusted away. Consequently, conventional linear adjustment for age may not be fully appropriate in this context and could potentially obscure true biological associations. Future studies should account for this age-related heterogeneity at the design stage, for instance, by stratifying patients based on age of onset or enrolling homogeneous cohorts within specific age ranges, to further validate the findings of this study.

## Data Availability

The datasets presented in this study can be found in online repositories. The names of the repository/repositories and accession number(s) can be found below: https://www.ncbi.nlm.nih.gov/, PRJNA1274372.
